# Underage and underserved: reaching young women who sell sex in Zimbabwe

**DOI:** 10.1080/09540121.2016.1176673

**Published:** 2016-07-08

**Authors:** Joanna Busza, Sibongile Mtetwa, Rumbidzo Mapfumo, Dagmar Hanisch, Ramona Wong-Gruenwald, Frances Cowan

**Affiliations:** ^a^ Department of Population Health, London School of Hygiene and Tropical Medicine, London, UK; ^b^ Centre for Sexual Health, HIV and AIDS Research (CeSHHAR), Harare, Zimbabwe; ^c^ United Nations Population Fund (UNFPA), 605 3rd Avenue, New York, NY, United States; ^d^ German Development Cooperation, Harare, Zimbabwe; ^e^ Department of Infection & Population Health, University College London (UCL), London, UK

**Keywords:** Key populations, sex work, Zimbabwe, adolescents, process evaluation

## Abstract

Young women who sell sex (YWSS) in Southern Africa are highly vulnerable to HIV, as the risks of being young and female in a high prevalence setting coalesce with those of commercial sex. YWSS are less able to negotiate safe sex, more likely to have higher risk partners, and less likely to use available health services compared to older sex workers. In Zimbabwe’s national HIV programme for sex workers, fewer than 1% of clients were 15–29. We developed monthly interactive workshops for YWSS based on an Activity Pack consisting of 21 sessions organised into six modules. The aim was to encourage YWSS’ interaction with each other, build their trust, confidence and skills, and encourage uptake of clinical services. We conducted a process evaluation to assess programme strengths, identify challenges, and recommend changes. This paper presents findings synthesising programme records with qualitative data and discusses feasibility, acceptability, and outputs during the pilot phase. In total, 143 YWSS attended meetings and most were from the target 15–19-year-old age group. Participants enjoyed the sessions and reported improved cooperation, willingness to negotiate with clients, and self-reflection about their futures. Staff found facilitating sessions easy and activities clear and appropriate. Challenges included identifying appropriate referrals, initial recruitment of women in some sites, and managing participants’ requests for financial compensation. The number of clients aged 15–19 increased at sex worker clinics in all sites. This programme is the first to target YWSS in Zimbabwe to address their disproportionately low service use. It proved feasible to staff and acceptable to participants over a one-year period. Given enhanced vulnerability of YWSS, this programme provides one workable model for reaching this underserved group.

## Introduction

Key populations experience disproportionately high HIV prevalence and are prioritised by the global HIV response (WHO, 2014). Comprising men who have sex with men, sex workers, people who inject drugs, and transgender people, key populations are not mutually exclusive, and many people experience overlapping risks, such as sex workers who inject drugs, or men who have sex with men and also sell sex (Okal et al., 2013; Rusakova, Rakhmetova, & Strathdee, 2014). Until recently HIV programmes targeting key populations have focused on adults but increasing attention is being drawn to those who are adolescents (13–19) or young people (15–24) (Lall, Lim, Khairuddin, & Kamarulzaman, 2015).

Younger key populations experience greater HIV risk than their older counterparts as structural barriers related to being young and engaging in specific risks coalesce, disadvantaging them for both HIV acquisition and service use (Delany-Moretlwe et al., 2015). Adolescents pose particular challenges as in most countries under-18s are classified as minors and often require parental permission for HIV testing and treatment. Child protection policies can further restrict programmatic options such as harm reduction (Curth, Hansson, Storm, & Lazarus, 2009; UNICEF, 2010).

Young women (15–24) who sell sex (YWSS) are one example of a key population for whom offering services is hampered by controversy and lack of clarity on how to meet their needs (Traore et al., 2015; UNAIDS, 2014). International agreements define YWSS under 18 as victims of trafficking and/or sexual exploitation, making many providers wary of legal repercussions (McClure, Chandler, & Bissell, 2014). Studies demonstrate YWSS are less able to negotiate safe sex, more likely to have higher risk partners, and less likely to use health services compared to older sex workers (Lau, Tsui, Siah, & Zhang, 2002; Sarkar et al., 2006; Shahmanesh et al., 2009).

As a result, young women are often exposed to HIV soon after they start selling sex, providing a brief window of opportunity to reach them with effective prevention, while those with HIV may delay testing and antiretroviral treatment (ART) (Goldenberg et al., 2012; McKinnon et al., 2015; Silverman, 2011). Women aged 15–24 often require other sexual and reproductive health services, as well as assistance with education, housing, parenting, mental health, and gender-based violence (Santos, 2009; USAID, 2012).

In Southern Africa, adolescent and young women are already at heightened risk for HIV, prompting calls to consider them a key population in their own right (Dellar, Dlamini, & Karim, 2015). Those who sell sex experience exacerbated vulnerability in this setting (Busza, Mtetwa, Chirawu, & Cowan, 2014; Cowan et al., 2013). Yet despite the large number of programmes for adolescent girls and young women in the general population and those targeting sex workers, few interventions have brought the aims, resources, and skills from these programmes together to address the needs of YWSS. This paper describes an attempt to do so through a one-year pilot programme delivered in three sites in Zimbabwe that focused on adolescent YWSS 15–19 and adopted a combined approach linking community mobilisation to clinical services as recommended by the literature on effective programming for sex workers and recent guidance specific to adolescent key populations (Bekker et al., 2014; Kerrigan, Fonner, Stromdahl, & Kennedy, 2013; UNAIDS, 2014).

## Background

The Centre for Sexual Health and HIV/AIDS Research (CeSHHAR) implements Zimbabwe’s national sex worker prevention and treatment programme on behalf of the National AIDS Council. The *Sisters with a Voice* (Sisters) programme started in 2009 and has reached over 24,000 female sex workers in 36 sites with clinical services, health education, and community mobilisation. Both fixed and mobile clinics provide primary health care, contraception, management of sexually transmitted infections, and HIV testing and referral for ART. Sisters’ counsellors, community outreach workers, and peer educators supplement clinical services with participatory activities aimed at building social cohesion, support networks, and self-efficacy among sex workers.

Despite success in engaging adults, reaching YWSS has proved challenging. In 2013 fewer than 1% of Sisters participants were under the age of 18. A respondent-driven sampling survey conducted by CeSHHAR among 2722 sex workers in 14 sites found younger women had significantly lower levels of HIV testing and engagement with care (Napierala Mavedzenge et al., 2015). Following receipt of funding to initiate a pilot intervention, CeSHHAR conducted a programme planning assessment. Initial exploration by Sisters outreach workers indicated a “hidden” population of YWSS both unaware of services and reluctant to use them. Fourteen YWSS, opportunistically recruited from among those ever-attending a Sisters clinic or community mobilisation meeting, participated in a one-day workshop to discuss their needs. Priority issues identified during this session included protection from violence, negotiating with clients, gaining confidence to use clinical services, and accessing education or income-generating opportunities.

The YWSS programme centred on interactive workshops developed through a review of existing materials for adult sex workers, vulnerable adolescent girls, and young people living with HIV. The first author brought together training and skills-building resources from her own collection, for example, Making Sex Work Safe (Network of Sex Work Projects, 1997) and additional materials obtained through online searches and requests to existing programmes working with adolescents or sex workers in southern Africa, including AIDSTAR, Peace Corps, UNFPA. Activities were adapted to fit the local Zimbabwean context and new ones designed. The Activity Pack consists of 21 activities organised into six thematic modules (Table 1).

**Table 1.** Activity Pack modules and activities.

Module	Activity title
Starting the Journey Together	Musical Chairs Conversation Starters Who are We? Setting Group Rules & CeSHHAR Project Principles
Friends & Peers	What makes a REAL friend? What is a Community? What are our Priorities?
Work & Play	I am a … Negotiation: Learning to get what you want! Supporting Each Other to Stay Safe at Work Staying Safe Outside Work
Caring for My Body	Knowing My Body Facts about Sexual and Reproductive Health Staying Healthy – Traffic Lights
Looking after Ourselves & Each Other	Overcoming Barriers to Seeking Help Tips for Problem Solving What is an Empowered Sex Worker? What is Community Mobilisation?
Planning for the Future	My Ideal Timeline Taking Responsibility for My Future Where can I find support? Guest Lecture Moving Forward

The aim of the Activity Pack was to encourage YWSS’ interaction with each other and with staff, build trust, confidence and skills, and encourage uptake of clinical services. Monthly meetings were held in each site, to which YWSS were invited through word of mouth. The 14 participants from the planning focus group were trained in basic peer education (building rapport, active listening, information provision, sexual and reproductive health) and assisted with recruitment. Young peer educators were 16–19 years old and had been selling sex for between 6 months and 2 years. The intervention included referral links to the Department of Labour and Social Services for child protection through their national programme for orphans and vulnerable children.

We conducted a process evaluation to assess the pilot’s strengths, identify challenges, and recommend changes. This paper presents findings synthesising programme records with qualitative data and discusses the feasibility, acceptability, and outputs following the pilot phase, as well as implications for future delivery.

## Process evaluation methods

Relevant measures in a process evaluation include *fidelity to design* (were activities conducted as planned?), *feasibility of delivery* (what challenges were faced and were they overcome?), *coverage* (who was reached and were they from intended target groups?), *acceptability* (how were activities perceived by participants and staff?), and *quality* (how well were activities delivered?). In some cases it may be possible to consider early signs of *effectiveness* (do activities appear to lead to predicted change?). Table 2 summarises how these measures were translated into evaluation questions.

**Table 2.** Process evaluation guiding questions.

Measure	Key questions
Fidelity	Were YWSS activities delivered as planned and scheduled? Was the Activity Pack used as designed?
Feasibility	What challenges were encountered in implementing the YWSS program, and specifically in using the Activity Pack?
Coverage	How many women were reached in each site? Were the participants from the target group of 15–19 YWSS?
Acceptability	What are YWSS perceptions of the programme? Do YWSS actively participate in Activity Pack sessions? Are YWSS able to attend meetings and services easily? What are staff members’ perceptions of the programme?
Quality	Are activities included in the Activity Pack appropriate for the target audience? Does the Activity Pack reflect evidence-based best practices for working with YWSS? Were Activity Pack sessions well conducted?
Effectiveness	Are intended outputs (uptake of services, referrals to other services, enhanced confidence and skills) occurring?

To answer these questions, the process evaluation draws on routine documentation and qualitative tools (Table 3). Structured interviews with the staff member most closely involved implementing the intervention in each site were conducted by the first author and focused on facilitators and barriers to introducing the activities and their overall feasibility. The second author conducted the monitoring and evaluation workshops. Observation of two sessions per site was conducted by the third author, an independent consultant with experience working with sex workers, using a standardised form for recording number of participants, issues raised, perceived comfort levels of both staff and YWSS, and any deviations from the activity description provided in the Activity Pack.

**Table 3.** Process evaluation methods.

Source	Indicators
Monthly staff reports	Frequency and timing of workshops Numbers of women attending sessions per site Activities conducted per site Referrals to other services
Observations by external consultant (two per site)	Emerging challenges Levels of participation Staff competence in conducting activities
Discussions with Staff	Activity Pack usefulness and ease of delivery Facilitators and barriers to delivering intervention Suggested improvements
Participatory M&E activity (one per site)	Perception of YWSS programme by participants Feedback on specific Activity Pack sessions Attitudes to Sisters programme
Aggregated clinic records	Numbers of YWSS attending clinical services since 2013

The participatory monitoring and evaluation activity consisted of three parts. First, YWSS worked in small groups to list all the activities they remembered, rank them in order of preference, and discuss what they liked, disliked, and would like to change. To explore opinions they may wish not to share, the facilitator read out the following statements, and women indicated their level of agreement using a 5-point Likert scale on an anonymous piece of paper:

1. It is easy for me to attend Sisters activities.
2. Sometimes it makes me uncomfortable to discuss the issues in the activities with others.
3. I have recommended the activities to other people.
4. The activities take too much time.
5. I have talked to others about what the topics we have discussed in these sessions in my own personal life.
6. I have gone to the Sisters Clinic at least once.

They were also asked for age, number of clients in the past week, and number of times each woman had attended a participatory session and Sisters clinic. Given the small number of women attending each workshop (11, 12, and 35 in Hwange, Mutare, and Victoria Falls, respectively) and the fact they were self-selected as volunteers, these data are indicative rather than representative. Finally, information on numbers of clients in the target age range was compiled from electronic patient records collected regularly as part of routine programme data.

## Results

### Delivery of intervention

The YWSS programme was introduced in January 2015 in Mutare, and February in Victoria Falls, and Hwange following training for field staff on the Activity Pack sessions. Most sessions lasted 60–90 minutes, including a warm-up activity to break down social barriers between participants who did not know each other. “Ice breakers” were followed by one to two individual or small group tasks that lead into wider discussions. Although the Activity Pack provided guidance on the order of activities, facilitators opted to select activities on an *ad hoc* basis depending on participant numbers and interests as well as facilitators’ comfort in delivering a specific activity. Over the 9 months covered by the process evaluation, 7 out of 21 sessions were conducted in Mutare, 9 in Victoria Falls, and 8 in Hwange. Observations found that facilitators were enthusiastic, delivered sessions as specified, and competently managed emerging questions and potential conflict.

In total, 143 YWSS attended meetings. The average number of participants per session was 15 in Mutare, 16 in Victoria Falls, and 13 in Hwange. The lowest turnout was in Mutare when three women attended and staff substituted the intended activity with a condom demonstration. The largest number of participants was in Victoria Falls with a total of 31 women. This was an unusually large group and was organised opportunistically while women were waiting to be transported to a meeting organised by a different organisation.

Accurate age can be difficult to elicit given sensitivities around underage sex work. Staff estimated most participants were 18 and 19 with some 16- and 17-year-olds, particularly in Victoria Falls and Hwange. During the participatory monitoring and evaluation sessions, the youngest age given was 15 and the oldest 24. In Mutare participants did not self-identify as selling sex and referred to themselves as “women with multiple boyfriends”. By contrast, YWSS in Victoria Falls and Hwange considered themselves professional sex workers and differentiated between clients and boyfriends.

Despite efforts by staff to sensitise local welfare officers to YWSS needs and forge constructive links, planned referrals to social services did not occur as YWSS feared being taken into care. Attempts to link two women to affordable abortion services also failed, although several women received assistance from staff to complete job applications or re-start education.

### Participant views

YWSS reactions to the programme were positive, although this is biased by self-selection of volunteers for the monitoring and evaluation session. Women could remember and describe most activities and had attended an average of 3 (range 0–10). In all sites, YWSS said they liked having been taught about their bodies and were more aware of their sexuality. The interactive workshops enhanced cooperation among YWSS so they were more likely to warn each other about violent clients, those who do not like using condoms, or do not pay. Awareness of their rights, improved trust of the broader Sisters programme, and increased self-confidence were also mentioned as workshop outcomes.

When asked about what they liked, YWSS mentioned Sisters clinics, appreciating that they were free and provided condoms, family planning, and sexually transmitted infections (STI) treatment. Many YWSS described staff as friendly and welcoming:

They treat us well here despite our age. At other facilities they will start asking questions like why are you doing this? and why you have an STI? or why do you want condoms at such a tender age? (Vic Falls)

Our nurse is good. Even when you get here when she’s going home, she attends to us. (Hwange)

The love we get from the staff here is awesome! (Mutare)

In all sites, “Planning for My Future” was voted as the favourite module. YWSS said the activity helped them think more seriously and there was agreement that most YWSS usually felt hopeless or lost when considering their future:

I used to be rowdy and not care about the future, I had no hope for the future because I thought as a sex worker that’s it until I had that session. (Victoria Falls)

On the other hand, all three groups stated they did not like the time management. Meetings started up to one hour late as facilitators waited for arriving participants. In Hwange and Victoria Falls, participants asked for refreshments to be provided and complained about lack of financial reimbursement. In Mutare, participants reported not being comfortable around a clinic known for serving sex workers. They did not want to be seen by older sex workers coming for treatment who might gossip about YWSS. A private room had been requested by staff, but was usually not available.

YWSS in Victoria Falls also did not like mixing with older sex workers:

[They] later disclose us in the community, telling everyone … “this child is a sex worker”.

One participant suggested that the older sex workers need to understand that they are all in the same trade despite age differences.

Results from the confidential Likert scale reflect YWSS satisfaction. Of 57 participants, 54 said they “agreed” or “agreed a lot” with the statement “It is easy for me to attend Sisters activities” and 43 with “I have recommended the activities to other people.” The majority also reported having talked to others about the topics raised (37) and have attended the clinic at least once (47). There was less agreement about whether the activities took too much time (25 thought so) or could make women feel uncomfortable (15 agreed, while 7 remained neutral).

### Staff views

The three interviewed staff members said facilitating sessions was straightforward and the Activity Pack clear, easy to follow, and relevant. One mentioned she was nervous to work with younger women but soon became comfortable and was touched by YWSS’ appreciation for “learning new things” and being listened to and cared about. Staff felt YWSS enjoyed activities, particularly those that involved singing and dancing. Staff also noted popularity of “Planning for My Future” which led to a deeper reflection than some of the more “fact-based” modules.

Yet staff highlighted that YWSS could be reluctant to attend sessions in Victoria Falls and Hwange. Although the total number of women reached and the average number of participants per activity were not much lower than in Mutare, staff in these sites had to use more proactive means to recruit YWSS, such as phone calls and home visits. The reasons for this were unclear but appeared related to YWSS’ feeling “busy” and not wanting to attend clinics unless needing medical services.

Staff reiterated participants’ reports of disliking to mix with adult sex workers. Younger women told staff that they are bullied and patronised by adult sex workers at bars and other locations where they sell sex, and wanted their own dedicated sessions. This also indicated that YWSS had lower clinic attendance because of services’ popularity with adult sex workers.

### Clinic attendance

One aim of the YWSS programme was to encourage Sisters clinic use. We compared numbers of new and all clients from 2013 through 2015 from data routinely collected at every visit. Among new clients, 15–19-year-olds increased to 28% in 2015 from 14% in 2013 and 18% in 2014.

For total number of client visits, in Hwange the proportion made by 15–19-year-olds was 3% in 2013, 2% in 2014, and 11% in 2015. In Victoria Falls, this was 0 in 2013, 16% in 2014, and 17% in 2015. In Mutare, YWSS made up 9% of all clinic visits in 2013, 21% in 2014, and 16% in 2015. The 2015 figure is skewed by the fact that clinic visits from all *age* groups increased substantially; in numbers, clinic visits by 15–19-year-olds in Mutare grew from 12 in both 2013 and 2014 to 76 in 2015.

These figures serve as a proxy measure for potential effect of the participatory sessions in reducing young women’s anxieties around using the clinics and concerted efforts by outreach workers to reach YWSS in each community. With the exception of Mutare, a similarly large increase in new clients was not seen for older age groups, strengthening the possibility that the workshops contributed to uptake, although lack of a rigorous outcome evaluation including comparison sites precludes attribution.

## Discussion

Our process evaluation suggests good *fidelity* as sessions were delivered monthly on schedule. While sessions were not ordered as intended, we encouraged facilitators to assess participants’ interests and select activities accordingly and do not believe this is likely to negatively affect the programme. Over time it will be important to ensure the full package of 21 activities is delivered. The programme’s *feasibility* is demonstrated by staff’s ease in conducting sessions. Despite concerns about the public location of the venues, participation levels remained consistent. In Victoria Falls and Hwange, however, more proactive means of recruiting YWSS were required. Although participation rates there were only slightly lower, intensive attempts to maintain interest are not sustainable. We hope greater trust will develop over time to increase YWSS’ motivation to participate, particularly as in future it may not be possible to wait for women to arrive, as this causes delays and frustration.

*Coverage* of YWSS cannot be determined without a population estimate. We reached 52 women in Mutare, 43 in Victoria Falls, and 48 in Hwange, all from our target age group, which is an extremely difficult demographic cohort to recruit. *Acceptability* among YWSS also appears good. The growing numbers of women attending workshops is further testimony to their appropriateness. The most commonly reported barrier to attendance included fear of being recognised by older sex workers, particularly at clinic sites.

As a measure of *quality*, observations of six sessions concluded they were delivered as designed. Staff found some activities easier to deliver and may need refresher training to ensure they confidently facilitate the more complicated sessions and maintain their skills.

Although a process evaluation cannot indicate *effectiveness*, the increase of 15–19-year-olds as a proportion of new and all clients suggests the YWSS programme may have increased uptake. During the pilot, outreach workers and the young peer educators made concerted efforts to reach YWSS in the community to inform them of the full range of Sisters services and not just the participatory workshops. The workshops themselves, however, were an opportunity to alert YWSS about the clinic and encourage them to spread the word among their social networks.

Numerous challenges remain. First, YWSS demonstrate a wide range of needs beyond HIV, including help in accessing educational and income-generating opportunities. CeSHHAR is not equipped to meet all YWSS’ complex needs and will need to find suitable referral services, which were not available during the pilot. YWSS’ anxiety around mixing with adult sex workers also poses a barrier to participation and as Sisters cannot offer parallel clinical services. The hope is that once YWSS develop greater confidence, they will be more open to mixing with adult sex workers. Sisters can also work to improve adult sex workers’ attitudes towards YWSS. Finally, YWSS are not a homogeneous group, exhibiting different levels of willingness to self-identify. We need to remain sensitive to YWSS’ own perceptions and motivations and adjust the language and tone of activities accordingly.

In future, we hope to refine and expand the YWSS programme, which has been selected as a site for the United States Agency for International Development-funded DREAMS initiative targeting high-risk adolescents and young women. This will allow for provision in new sites, experimentation with location and timing of meetings, and training of additional young peer educators who may be able to help facilitate sessions over time. Existing and new peer educators will be further trained in the process of micro-planning to help identify the most vulnerable YWSS in each community and ways to engage them (Bill & Melinda Gates Foundation, 2013). The DREAMS package will also introduce educational subsidies and income-generating opportunities to which we will be able to refer YWSS, and provide the opportunity to conduct an impact-level evaluation.

## Conclusion

CeSHHAR’s YWSS programme represents the first time this group has been targeted through social and clinical services in Zimbabwe. Following a one-year pilot programme to recruit, engage and link YWSS to existing prevention and treatment, the programme proved feasible to staff and acceptable to participants, reached close to 150 women from an extremely hard-to-reach group, and may have increased numbers of women attending clinical services. Given global acknowledgement of enhanced vulnerability among young key populations, this programme provides one workable model for reaching this underserved group.
